# Supplementary material for: Identification of photoperiod-induced specific miRNAs in the adrenal glands of Sunite sheep (Ovis aries)
Source: Front Vet Sci. 2022 Jul 22;9:888207. doi: 10.3389/fvets.2022.888207 (PMC9354845; doi:10.3389/fvets.2022.888207)
Supplement: Supplementary file 1 [file Data_Sheet_1.ZIP › Supplementary file/miRDeep2 file.zip!/miRDeep2 file/Novel_Structure/SPLP42A3/Novel_336.pdf]

Provisional ID	:	NC_019463.2_14496
Score total	:	1.6
Score for star read(s)	:	-1.3
Score for read counts	:	0
Score for mfe	:	1.3
Score for randfold	:	1.6
Score for cons. seed	:	
Total read count	:	815
Mature read count	:	814
Loop read count	:	0
Star read count	:	1

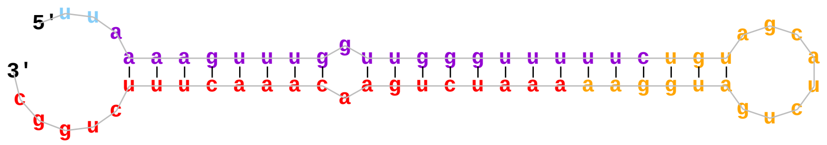

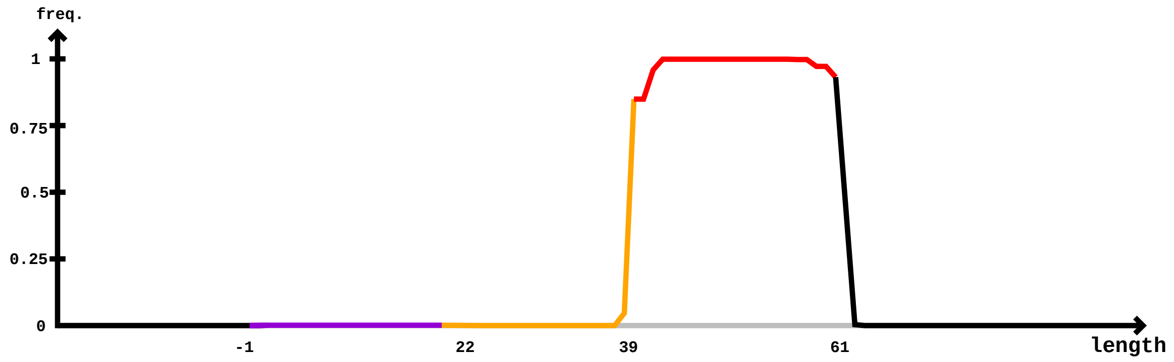

Star **Mature**

```
5'- augaaacauuggauuggcuuaaaaguuugguuggguuuuucuguagcaucugauggaaaaaucugaacaaacuuucuggccaacucauugguuguaucuugcugcucaaga   -3'    obs
    augaaacauuggauuggcuuaaaaguuugguuggguuuuucuguagcaucugauggaaaaaucugaacaaacuuucuggccaacucauugguuguaucuugcugcucaaga          exp
    ......((.(((.((((((..((((((((.((((((((((((((........)))))))))))))).))))))))..)))))).))).))......(((((.....)))))   reads   mm   sample
    ....................aaaaguuugguuggguuuuuc......................................................................   1       0    seq
    .........................................................aaaaucugaacaaacuuu....................................   1       0    seq
    .........................................................aaaaucugaacaaacuuuUu..................................   18      1    seq
    .........................................................aaaaucugaacaaacuuuUugg................................   19      1    seq
    ..........................................................aaaucugaacaaacuuuUu..................................   3       1    seq
    ..........................................................aaaucugaacaaacuuuUugg................................   14      1    seq
    ..........................................................aaaucugaacaaacuuuUuggc...............................   637     1    seq
    ............................................................aucugaacaaacuuuUuggc...............................   16      1    seq
    ............................................................aucugaacaaacuuuUuggcc..............................   73      1    seq
    .............................................................ucugaacaaacuuuUuggcc..............................   30      1    seq
    .............................................................ucugaacaaacuuuUuggcca.............................   3       1    seq
```
